# Long-term survivorship of a medial-pivot total knee system compared with other cemented designs in an arthroplasty registry

**DOI:** 10.1186/s13018-016-0388-8

**Published:** 2016-04-19

**Authors:** Barbara Bordini, Cristina Ancarani, David A. Fitch

**Affiliations:** Istituto Ortopedico Rizzoli, Bologna, Italy; MicroPort Orthopedics Inc., 5677 Airline Rd., Arlington, TN 38002 USA

**Keywords:** Medial-pivot, Registry, Total knee replacement, Total knee arthroplasty

## Abstract

**Background:**

The Orthopaedic Data Evaluation Panel (ODEP) monitors the performance of primary total knee arthroplasty (TKA) implants against guidance provided by the National Institute for Health and Care Excellence (NICE) and issues ratings based upon survivorship data meeting or exceeding 95 % at 10-year follow-up. The objectives of the current study were to determine if the survivorship for the ADVANCE Medial-Pivot System in an arthroplasty registry exceeds this threshold and if its survivorship is significantly different than that of all other cemented bi-, tricompartmental, minimally stabilized, and fixed bearing TKAs in the same registry.

**Methods:**

The database of an arthroplasty registry was searched for all TKAs performed with the subject system and all other cemented TKAs. The Kaplan-Meier survivorship for the subject system was compared to the NICE criteria and also that of all other cemented TKAs. Complication modes were also examined for the two groups.

**Results:**

The 10-year survivorship for the included 506 TKAs using the subject system (96.3 %) exceeded the NICE guidelines of 95 % at 10 years. Survivorship also exceeded that of all other cemented TKAs (95.7 %) in the same registry, but the difference was not significantly different.

**Conclusions:**

The subject system was associated with survivorship that exceeds the NICE guidelines at 10 years and is similar to that of other cemented TKA systems. The use of this unique tibial insert design does not negatively impact component survivorship.

## Background

The Orthopaedic Data Evaluation Panel (ODEP) in the UK was established in 2002 to monitor the performance of primary total hip arthroplasty (THA) implants against guidance provided by the National Institute for Health and Care Excellence (NICE) [1]. The NICE guidelines initially stated that only THA implants with cumulative survivorship of at least 90 % at 10-year follow-up should be used in clinical practice [2]. In 2014, the recommended survivorship was increased to at least 95 % at 10 years based upon an updated review of available evidence [3]. While the NICE has not yet issued a technology appraisal for total knee arthroplasty (TKA) implants, the ODEP recently began reviewing these implants and basing ratings upon the survivorship guidelines for THA. Recent annual reports for the Australian Orthopaedic Association National Joint Replacement Registry and the National Joint Registry for England, Wales, Northern Ireland, and the Isle of Man reported 10-year survivorship for all cemented TKAs of 94.8 and 96.6 %, respectively [4, 5]. This suggests it is likely reasonable for the ODEP to use the THA criteria as the basis for its TKA ratings. These ratings and, by extension, survivorship are critically important, as some hospitals are now requiring a minimum rating before a device can be implanted by their surgeons.

The ADVANCE® Medial-Pivot System (MicroPort Orthopedics Inc., Arlington, TN, USA) features a unique asymmetrical tibial insert that seeks to closely mimic the kinematics of the natural knee [6, 7]. Recently published single-center studies using this system have reported satisfactory midterm (5–8 years) survivorship estimates [8–10,] and a recent meta-analysis containing data from eight studies and over 1100 TKAs reported 99.2 % and 97.6 % survivorship at 5 and 8 years, respectively [11]. While this system has been on the market for over 17 years, there have yet to be any published reports of long-term survivorship using it. The primary objective of this study was to determine if the long-term survivorship of the ADVANCE Medial-Pivot System in an arthroplasty registry exceeds 95 % at 10 years follow-up. The secondary objective was to determine how the survivorship for this system compared to all other cemented bi-, tricompartmental, minimally stabilized, and fixed-bearing TKAs in the same registry.

## Methods

The database for the Register of Orthopaedic Prosthetic Implants (RIPO) was searched for all TKAs implanted with the ADVANCE Medial-Pivot System identified by product code between July 1, 2000 and December 31, 2013. The RIPO was established at the Istituto Ortopedico Rizzoli in the Emilia-Romagna region of Italy and collects data related to all TKAs and THAs performed on the region’s nearly 4.5 million residents [12]. All primary TKAs implanted with the subject system were included in the analysis. TKAs were excluded if they were implanted in patients who lived outside of the Emilia-Romagna region to minimize bias due to loss to follow-up. For residents of the Emilia-Romagna region, any treatment received in other regions of Italy is billed back to the Emilia-Romagna region and therefore captured in the registry. For residents of other regions that had their TKA performed in the Emilia-Romagna region, future treatment received outside of the Emilia-Romagna region is billed to their home region and therefore not captured in the registry. Due to these limitations of the registry and healthcare system, the most accurate and simplest method to minimize bias and not overestimate survivorship was to use only data from the Emilia-Romagna region. For the same time period, data was also retrieved for all other bi-, tricompartmental, minimally stabilized, and fixed-bearing cemented TKAs to serve as a comparison group. Ethics approval was not necessary as the data was collected from a registry in Italy that collects data as standard practice on all patients in their region. Additionally, all data was collected and analyzed in a de-identified format that protects patient privacy.

### Statistical considerations

For both cohorts, subject demographics and reasons for revision were presented as a percentage of the total cohort. Patient age and body mass index were compared using a *t* test (*p* < 0.05), while gender and indication for surgery were compared using chi-square analysis (*p* < 0.05). Kaplan-Meier survivorship analysis was performed using revision of any component as the endpoint and survival times of unrevised TKAs taken as the last date of observation (December 31, 2013 or date of death). The log-rank test was used to compare survivorship between the two groups. The Cox multiple regression model for analyzing survival data was considered. The proportionality hazards assumption was tested by the Schoenfeld residual method; age and gender used for adjustment fulfilled the proportional hazard assumption for the all period.

The Wald test was used to calculate the *p* values for data obtained from the Cox multiple regression analyses.

Differences between groups were considered statistically significant if the *p* values were less than 0.05. All statistical analyses were performed using SPSS software (version 14.0.1, Chicago, Illinois).

## Results

There were 506 TKAs performed with the subject system by 30 orthopedic surgeons at more than ten hospitals during the time period of interest. A large majority of patients were female (72.3 %), and over 70 % were considered overweight or obese (Table 1). All components were implanted with cemented fixation, and the patella was resurfaced in only three (0.6 %) TKAs. The mean follow-up was 6.6 years (range, 0.02–13.0), and there were no intraoperative complications reported for any patient during hospitalization. Demographics for the 20,446 TKAs included in the All Other Cemented TKAs group were similar to those of the ADVANCE group (Table 1). There were no statistical differences in body mass index, age, or gender between the two groups. Patients in the All Other Cemented TKAs group were significantly younger (*p* = 0.001).

**Table 1:** Demographics for patients in the two study groups

	ADVANCE	All Other Cemented TKAs
Mean age (years)	73.4 (24–90)	71.7 (24–92)
Male (%)/female (%)	27.7 %/72.3 %	29.0 %/71.0 %
Body mass index		
Underweight (≤19)	0.2 %	0.2 %
Normal (20–25)	27.8 %	19.3 %
Overweight (26–29)	45.5 %	46.4 %
Obese (≥30)	26.4 %	34.1 %
Indication for TKA		
Primary arthritis (%)	89.9 %	87.1 %
Deformity (%)	3.4 %	6.9 %
Rheumatoid arthritis (%)	2.4 %	1.3 %
Post-traumatic arthritis (%)	2.2 %	1.2 %
Chondrocalcinosis (%)	1.0 %	1.0 %
Sequelae of osteotomy (%)	0.6 %	0.8 %
Other (%)	0.6 %	1.7 %

**Fig. 1:**
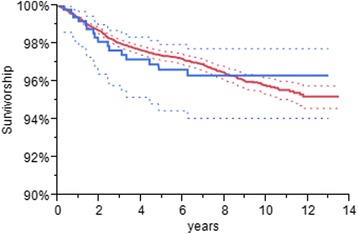
Survivorship estimates for the ADVANCE (*blue line*) and the All Cemented TKAs (*red lines*) are shown with their associated 95 % confidence intervals

The Kaplan-Meier survivorship estimate at 10 years was 96.3 % (95 % CI, 94.5–98.1) for the ADVANCE group (Table 2). In this table, N at Risk refers to the number of TKAs that were available at the beginning of each year interval. This was higher than the 10-year rate of 95.7 % (95 % CI, 95.3–96.2) for the All Other Cemented TKAs group (Table 3), but the difference was not statistically significant (Fig. 1). There were 16 (3.1 %) revisions in the ADVANCE group: 9 for aseptic loosening where both the tibial and femoral components were revised; 5 for septic loosening; and 2 for aseptic loosening where only the tibial component was revised (Table 4). The adjusted risk of revision was not significantly different for the ADVANCE and All Other Cemented TKAs groups. (*p* = 0.662). Results of the Cox regression analysis showed age at the time of surgery was the only variable influencing the risk of revision for any cause, with the risk of revision decreasing as the age at the time of surgery increased.

**Table 2:** Survivorship estimates and numbers at risk for the ADVANCE group

Year	Survivorship (%)	Lower 95 % CI	Upper 95 % CI	N Revisions	N Deaths	N At Risk
0	100.0	100.0	100.0	1	7	506
1	99.2	98.4	100.0	5	4	494
2	98.1	96.9	99.3	5	7	459
3	97.6	96.3	99.0	2	10	424
4	97.2	95.6	98.7	0	8	397
5	96.6	94.9	98.3	2	9	367
6	96.6	94.9	98.3	0	10	334
7	96.3	94.5	98.1	1	16	290
8	96.3	94.5	98.1	0	12	230
9	96.3	94.5	98.1	0	12	173
10	96.3	94.5	98.1	0	4	113
11	96.3	94.5	98.1	0	1	58
12	96.3	94.5	98.1	0	0	18
13	96.3	94.5	98.1	0	0	1

**Table 3:** Survivorship estimates and numbers at risk for the All Other Cemented TKAs group

Year	Survivorship (%)	Lower 95 % CI	Upper 95 % CI	N Revisions	N Deaths	N At Risk
0	100.0	100.0	100.0	122	163	20,446
1	99.4	99.3	99.5	119	181	19,342
2	98.7	98.5	98.8	102	234	17,070
3	98.0	97.8	98.2	47	253	14,890
4	97.6	97.4	97.9	32	249	12,799
5	97.4	97.1	97.6	15	246	10,787
6	97.2	96.9	97.5	23	232	8801
7	96.9	96.6	97.2	23	219	6931
8	96.4	96.1	96.8	20	190	5317
9	96.0	95.6	96.4	6	138	3950
10	95.7	95.3	96.2	4	112	2776
11	95.5	95.1	96.0	4	63	1835
12	95.2	94.6	95.8	0	36	1060
13	95.2	94.6	95.8	0	4	436

**Table 4:** Reasons for revision for the ADVANCE and All Other Cemented TKAs groups

Reason for revision	ADVANCE Medial-Pivot		All Other Cemented TKAs	
	*N*	%	*N*	%
Aseptic loosening—both components revised	9	1.77	150	0.73
Septic loosening	5	0.98	141	0.69
Aseptic loosening—only tibia revised	2	0.39	42	0.21
Pain without loosening	0	0	67	0.33
Liner wear	0	0	22	0.11
Aseptic loosening—only femur revised	0	0	15	0.07
Instability	0	0	15	0.07
Dislocation	0	0	14	0.07
Bone fracture	0	0	13	0.06
Breakage of prosthesis	0	0	5	0.02
Unknown	0	0	15	0.07
Other	0	0	18	0.09

## Discussion

The current study is the first to report long-term survivorship in patients implanted with the ADVANCE Medial-Pivot System. The 10-year component survivorship of 96.3 % for this large series of patients exceeded the 95 % threshold recommended by the NICE in the UK. This outcome was expected as the existing midterm publications for this system show survivorship estimates that are equivalent to or exceed the thresholds at 5 (97.5 %) and 7 (96.5 %) years follow-up [8–10, 13]. The current results are particularly encouraging in that they were collected by 30 surgeons at over ten centers and independently analyzed by an arthroplasty registry.

Between the late 1990s and the early 2000s, several studies examined the kinematics of the “natural knee” [14–16]. These studies concluded that throughout flexion, the medial condyle experiences very little antero-posterior motion while the lateral condyle moves in a pivot motion around the medial condyle. Komistek et al. showed that this motion was observed during several common activities including deep knee bends, chair rise, chair sit, and level walking [16]. The subject system features a unique tibial insert design that constrains motion in the medial compartment while allowing unrestricted motion in the lateral compartment. The tibial insert also features anterior and posterior lips, which seek to provide enhanced anterior-posterior stability by substituting for both the anterior and posterior cruciate ligaments. Fluoroscopic analysis has shown that this design results in motion that is similar to that of the natural knee described previously [6, 7]. It has also been associated with increased quadriceps efficiency and reduced compensation in contralateral legs when compared to patients implanted with posterior-stabilized tibial insert designs during inclined walking [17] and sit-to-stand tasks [18]. Another report showed that patients with bilateral TKAs preferred the subject system over posterior-stabilized, mobile-bearing, and posterior cruciate ligament retaining TKA systems implanted in the contralateral knee [19]. The author attributed this preference to the design providing anterior-posterior stability and in turn, an increased feeling of stability.

While the subject design has been shown to closely replicate the natural motion of the knee and be preferred over some implant designs, a recent publication has speculated that the anterior region of the insert may experience additional stress because the anterior motion of the medial femoral condyle is suppressed only by the geometry of the insert [6]. If increased stresses existed, they could manifest themselves as failures due to increased insert wear, instability, insert breakage, or loosening. To determine if this unique design does result in reduced long-term outcomes, the survivorship for the system was compared to that for all other cemented TKAs. The survivorship for the subject system was not significantly different than that reported for All Other Cemented TKAs in the RIPO, suggesting this design does not negatively impact longevity when compared to other available systems.

### Limitations

There are several limitations to the current study. First, the study design is retrospective and the data source is a regional registry, meaning data might not be applicable to other geographic regions or cultures (e.g., Asian cultures which perform more deep flexion activities). Second, the only endpoint analyzed is survivorship. While the ODEP only analyzes survivorship and study quality in their assessments of devices, other outcomes (e.g., functional outcomes, satisfaction) are needed to determine if a procedure should be considered a success for a patient. Third, the survivorship for the subject system exceeded the NICE guideline of 95 %, but the lower bound of the confidence interval (94.5 %) was slightly less. This suggests there is a slight possibility that the actual survivorship could be below the NICE guideline. Fourth, the ASA-status or other measure of functional status was not available as part of this dataset. Finally, the registry only captures postoperative complications that resulted in revision. This could lead to an underestimation of complications that occurred but did not require revision (e.g., infections).

## Conclusions

Results from the current study of over 500 TKAs show that component survivorship at 10 years for the subject system exceeds the NICE guidelines. Survivorship was also similar to other cemented bi-, tricompartmental, minimally stabilized, and fixed-bearing designs, suggesting that the use of this unique tibial insert design does not adversely impact outcomes when compared to other designs.

## References

[1] Orthopaedic Data Evaluation Panel (ODEP). http://www.odep.org.uk/Home.aspx. Accessed 16 June 2015.

[2] NICE (2002). Technology Appraisal Guidance TA44: guidance on the use of metal on metal hip resurfacing arthroplasty.

[3] NICE (2014). Technology Appraisal Guidance 304: total hip replacement and resurfacing arthroplasty for end-stage arthritis of the hip.

[4] Australian Orthopaedic Association National Joint Replacement Registry (2015). Annual report.

[5] National Joint Registry for England, Wales, Northern Ireland and the Isle of Man (2015). 12th Annual Report.

[6] Miyazaki Y, Nakamura T, Kogame K, Saito M, Yamamoto K, Suguro T (2011). Analysis of the kinematics of total knee prostheses with a medial pivot design. J Arthroplasty.

[7] Schmidt R, Komistek RD, Blaha JD, Penenberg BL, Maloney WJ (2003). Fluoroscopic analyses of cruciate-retaining and medial pivot knee implants. Clin Orthop Relat Res.

[8] Chinzei N, Ishida K, Tsumura N, Matsumoto T, Kitagawa A, Iguchi T, Nishida K, Akisue T, Kuroda R, Kurosaka M (2014). Satisfactory results at 8 years mean follow-up after ADVANCE(R) medial-pivot total knee arthroplasty. Knee.

[9] Karachalios T, Roidis N, Giotikas D, Bargiotas K, Varitimidis S, Malizos KN (2009). A mid-term clinical outcome study of the advance medial pivot knee arthroplasty. Knee.

[10] Vecchini E, Christodoulidis A, Magnan B, Ricci M, Regis D, Bartolozzi P (2012). Clinical and radiologic outcomes of total knee arthroplasty using the advance medial pivot prosthesis. A mean 7 years follow-up. Knee.

[11] Fitch DA, Sedacki K, Yang Y (2014). Mid- to long-term outcomes of a medial-pivot system for primary total knee replacement: a systematic review and meta-analysis. Bone Joint Res.

[12] Register of Orthopaedic Prosthetic Implants Annual Report. https://ripo.cineca.it. Accessed 9 Jan 2015.

[13] Anderson M, Kruse R, Leslie C, Levy L, Pritchett J, Hodge J (2010). Medium-term results of total knee arthroplasty using a medially pivoting implant: a multicenter study. J Surg Orthop Adv.

[14] Freeman MA, Pinskerova V (2005). The movement of the normal tibio-femoral joint. J Biomech.

[15] Iwaki H, Pinskerova V, Freeman MA (2000). Tibiofemoral movement 1: the shapes and relative movements of the femur and tibia in the unloaded cadaver knee. J Bone Joint Surg.

[16] Komistek RD, Dennis DA, Mahfouz M (2003). In vivo fluoroscopic analysis of the normal human knee. Clin Orthop Relat Res.

[17] Czyrnyj C, Reynolds S, Dervin G, Lamontagne M (2015). Muscle activation patterns during inclined gait: comparison between medial pivot and posterior stabilized knee prostheses. European Federation of National Associations of Orthopaedics and Traumatology 16th Congress, Prague, CZ.

[18] Czyrnyj C, Reynolds S, Dervin G, Lamontagne M (2015). Muscle activation and functional performance following TKA: a comparison between prosthetic designs. 2015 Canadian Orthopaedic Association Annual Meeting, Vancouver, BC, Canada.

[19] Pritchett JW (2011). Patients prefer a bicruciate-retaining or the medial pivot total knee prosthesis. J Arthroplasty.

